# *Lactarius deliciosus* Extract from Green Microwave-Assisted Eutectic Solvent Extraction as a Therapeutic Candidate Against Colon Cancer

**DOI:** 10.3390/antiox14121452

**Published:** 2025-12-02

**Authors:** Seyed Hesamoddin Bidooki, Beatriz Rodríguez-Martínez, Javier Quero, Luis Vicente Herrera-Marcos, Mónica Paesa, Marina Delgado-Machuca, Oscar F. Beas-Guzmán, Jesús Osada, Pedro Ferreira-Santos, María Jesús Rodríguez-Yoldi

**Affiliations:** 1 Department of Biochemistry and Molecular and Cellular Biology, Faculty of Veterinary Medicine, Health Research Institute of Aragon, University of Zaragoza, 50013 Zaragoza, Spain; sbidooki@unizar.es (S.H.B.); josada@unizar.es (J.O.); 2 Department of Chemical Engineering, Faculty of Science, University of Vigo (Campus Ourense), As Lagoas, 32004 Ourense, Spain; beatriz.rodriguez@uvigo.es; 3 Instituto de Agroecoloxía e Alimentación (IAA), University of Vigo (Campus Auga), As Lagoas, 32004 Ourense, Spain; 4 Department of Pharmacology and Physiology, Legal and Forensic Medicine, Faculty of Veterinary Medicine, Aragon Health Research Institute, University of Zaragoza, 50013 Zaragoza, Spain; javierquero94@gmail.com (J.Q.); lherrera@unizar.es (L.V.H.-M.); 5 Department of Chemical Engineering, University of Zaragoza, Campus Río Ebro-Edificio I+D, C/Poeta Mariano Esquillor S/N, 50018 Zaragoza, Spain; monicapaesamorales@gmail.com; 6 Institute of Nanoscience and Materials of Aragon (INMA), Consejo Superior de Investigaciones Científicas (CSIC), University of Zaragoza, 50009 Zaragoza, Spain; 7 Aragon Health Research Institute (IIS Aragon), 50009 Zaragoza, Spain; 8 Department of Molecular Medicine, School of Medicine, University of Colima, Colima 28040, Mexico; karla_machuca@ucol.mx (M.D.-M.); oscar.beas.11@gmail.com (O.F.B.-G.); 9 Agri-Food Institute of Aragon (IA2), Centro de Investigación y Tecnología Agroalimentaria de Aragón (CITA), University of Zaragoza, 50013 Zaragoza, Spain; 10 Biomedical Research Network Center for the Pathophysiology of Obesity and Nutrition (CIBEROBN), Carlos III Health Institute, 28029 Madrid, Spain

**Keywords:** *Lactarius deliciosus*, NaDES-microwave extraction, colon cancer, antioxidant, antiproliferative, antimetastatic, anti-inflammatory, Caco-2

## Abstract

*Lactarius deliciosus* is a widely distributed edible mushroom valued as a functional food due to its rich content of nutrients, phenolic compounds, and flavonoids, which contribute to its strong antioxidant and antimicrobial properties. The present study aimed to optimize a green microwave-assisted extraction method for maximal recovery of bioactive phenolic compounds from *Lactarius deliciosus* extract (LDE) and to evaluate its antioxidant, antiproliferative, antimetastatic, and anti-inflammatory effects on human colon carcinoma (Caco-2) cells. The study demonstrated that solvent polarity and composition critically influence the recovery of antioxidant biomolecules, identifying water and NaDES 1 (glycerol/glycine/water) as the most efficient and sustainable solvents for microwave-assisted extraction at 225 °C. The LDE showed high levels of phenolic compounds—particularly 4-hydroxybenzoic and vanillic acids—indicating potent antioxidant potential and possible anticancer efficacy. The results revealed that the LDE significantly reduced colony formation and cell adhesion in a dose-dependent manner, leading to nearly complete inhibition of clonogenic survival at the IC_50_ concentration and a marked increase in cell death among non-adherent colon cancer cells. In addition, LDE inhibited the proliferation of Caco-2 cells by inducing G0/G1 cell cycle arrest and apoptosis, associated with altered mitochondrial potential and increased caspase-3 activity. The LDE modified the redox balance of the cell by decreasing the ROS levels and exerts anti-inflammatory effects through significant downregulation of *NOS2* expression, without adversely affecting the intestinal barrier. The study concludes that LDE bioactive compounds show strong promise as anticancer and functional ingredients, demonstrating antioxidant, antiproliferative, antimetastatic, and anti-inflammatory effects.

## 1. Introduction

Historical trends demonstrate a mounting interest and research endeavors focused on the utilization of nutraceutical products derived from plants and fungi, with the objective of enhancing health and well-being due to their nutritional properties and biological potential [1]. Mushrooms have long been the focus of scientific inquiry due to their nutritional richness and biological potential, making them a subject of study in both the dietary and medicinal domains [2,3]. These bioresources have been demonstrated to provide high-quality proteins, vitamins, minerals, and fibers. They are also characterized by low caloric content and the absence of starch, which renders them suitable for regular consumption and as meat substitutes [2,3]. These bioresources have been shown to possess nutritional values comparable to those of vegetables [2,3].

Beyond their nutritional function, many mushrooms exhibit therapeutic properties [1,2,4,5]. A substantial body of research has evidenced their antioxidant, anti-inflammatory, antimicrobial, antihyperglycemic, immunomodulatory, antiproliferative, and anticancer properties in numerous cancers, including sarcoma, lung, epithelial, and colon cancer [5,6,7,8]. These findings support the consideration of mushrooms as promising sources of bioactive agents in cancer therapy. It has been demonstrated that bioactive extracts of species such as *Lactarius deliciosus*, *Coprinus comatus*, *Cantharellus cibarius*, and *Lycoperdon perlatum* have the capacity to inhibit the viability of glioblastoma cells [8].

*Lactarius deliciosus* (*L. deliciosus*), a species of the Russulaceae family, is widely consumed for its characteristic taste and texture and is distributed across tropical, subtropical, and temperate forests [7,8]. It is classified as a functional food, providing essential nutrients and non-nutrients such as phenolic compounds and flavonoids, along with robust antioxidant and antimicrobial activity [9]. A multitude of studies have indicated that *L. deliciosus* displays a protective effect in various pathological conditions, including cancer, thereby underscoring its potential for therapeutic applications [4,5,6,7,8,9,10]. While numerous mushrooms have been the focus of extensive research, there is a paucity of evidence regarding the anticancer effects of *L. deliciosus* in colorectal cancer.

Colorectal cancer is a major global health concern. Its incidence has been increasing at a precipitous rate, particularly among younger adults [11]. By 2030, estimates indicate a 90% and 124% increase in colon and rectal cancer cases, respectively, among individuals aged 20–34 years [12]. Mortality is primarily associated with metastasis, and despite the existence of chemotherapeutic regimens, such as taxanes, fluorouracil, cyclophosphamide, anthracyclines, and cisplatin, treatment efficacy remains limited [13]. These therapeutic interventions frequently result in adverse effects, damage normal cells, and enhance drug resistance in patients [13,14].

Therefore, the present study aimed to (i) evaluate different “green” solvent mixtures and their combination with microwave-assisted extraction to improve the recovery of bioactive phenolic compounds, and (ii) assess the antioxidant, antiproliferative, antimetastatic, and anti-inflammatory effects of *L. deliciosus* extract (LDE) on human colon carcinoma cells (Caco-2).

## 2. Materials and Methods

### 2.1. Material Collection

The fruiting bodies of *L. deliciosus* were collected in a mixed forest stand of pine and oak from Albarracin (Teruel, Spain) and were authenticated by Rubén Escribano and Fernando Martínez-Peña from the Centro de Investigación y Tecnología Agroalimentaria de Aragón (CITA, Zaragoza, Spain). The size of the fruiting bodies collected was similar, and they had not yet reached full maturity. The samples were subjected to freeze-drying for a period of four consecutive days, resulting in a final humidity level of 4%. This procedure was crucial to ensure the effective inactivation of spores and prevent the contamination or proliferation of microorganisms. Subsequently, the mushrooms were ground into a powder with a diameter smaller than 0.45 µm.

### 2.2. Extraction Conditions and Extract Preparation

After preparing the *L. deliciosus* powder, the next step involved selecting the most efficient solvent for extracting its bioactive phenolic compounds. To achieve this, a conventional solid–liquid extraction was performed using six different solvents: four natural deep eutectic solvents (NaDES), water, and two organic solvents (ethanol at 50% and 96% (*v*/*v*), and methanol at 99% (*v*/*v*)).

The extraction was carried out under controlled conditions with a solid–liquid ratio (SLR) of 1:10, at 50 °C for 2 h, using a shaker set at 200 rpm. The choice of the most suitable solvent was based on the antioxidant activity of the resulting *L. deliciosus* extracts (LDEs), which was evaluated through the ABTS and FRAP assays. These complementary methods are widely recognized as reliable indicators of phenolic and other redox-active compounds contributing to the antioxidant potential of the extracts.

The NaDES were selected based on their biocompatibility, non-toxicity, environmental sustainability, and potential efficiency in extracting phenolic compounds [15]. The formulations of the selected NaDES are presented in Table 1.

The NaDES were prepared in a molar ratio that is reported in Table 1. They were prepared in sealed flasks that contained a magnetic stirring bar. The mixtures were stirred and heated at 80 °C until homogeneous and transparent liquids were formed. Before the initiation of the extraction process, a water content of 30% was incorporated into the NaDES to reduce viscosity and thereby enable the diffusion of bioactive compounds [16].

Following the identification of the optimal solvents (NaDES 1 and water), microwave-assisted extraction (MAE) was applied to increase the efficiency of phenolic compound recovery and minimize processing time. Extractions were carried out in 30 mL Pyrex vessels using a Monowave 450 single-mode microwave reactor (Anton Paar Spain S.L.U., Madrid, Spain) operating at 850 W and equipped with an infrared temperature detector. Extractions are conducted at different temperatures (100, 150, 200 and 225 °C) for 5 min, with an SLR of 1:10. During the extraction process, the samples were subjected to magnetic stirring at a rate of 600 rpm to ensure the uniform distribution of the constituents within the sample.

### 2.3. Determination of Phenolic Content and Composition

Total phenolic content (TPC) was analyzed by the Folin–Ciocalteu colorimetric assay, using 10 µL of LD extract. Gallic acid was used as a standard compound (50–1500 mg/L, R^2^ = 0.998), and the data were expressed as milligrams of gallic acid equivalents (GAEs) per gram of dry *L. deliciosus* (mg GAE/g LD) [17].

Total flavonoid content (TFC) was analyzed by the aluminium chloride-based colorimetric assay, using rutin as a standard compound (10–800 mg/L, R^2^ = 0.997), and results were expressed as mg of rutin equivalents/g of RBP (mg RE/g RBP) [17].

The analysis of individual phenolic compounds in the extract was performed using high-performance liquid chromatography–electrospray ionization-mass spectrometry (HPLC-ESI-MS). The identification and quantification of these compounds were conducted in an Agilent 1260 series HPLC (Palo Alto, CA, USA), equipped with an AB SCIEX Triple Quad 3500 detector (Foster City, CA, USA), which utilizes an electrospray ionization (ESI) source. This analysis method was developed based on the methodology established by Jaouhari et al. [17]. The analysis was performed by injecting 5 µL of the extract into a Luna C18 column (150 mm × 2 mm; 3 µm particle diameter) from Phenomenex. The separation process involved the use of 0.1% formic acid (solvent A) and acetonitrile with 0.1% formic acid (solvent B) as eluents in a gradient (98% of A from 0 to 4.0 min, 98–80% of A from 4.0 to 7.0 min, 80–10% of A from 7.0 to 14.0 min, 10% of A from 14.0 to 15.0 min, 10–98% of A from 15.0 to 17.0 min) at a flow of 0.3 mL/min. A positive/negative ionization source with turbo V™ (ion spray voltage of 4500 V), with nitrogen as nebulizer and collision gas, was employed at a source temperature of 400 °C. The acquisition of the data was facilitated by employing multiple reaction monitoring (MRM) in conjunction with Analyst 1.6.2 software (AB Sciex, Foster City, CA, USA). The identification of each individual phenolic compound was determined by taking into account the retention time (RT), mass spectra (*m*/*z* of the ions), and the concentration was accessed using the calibration curve of each corresponding standard (Table S1, Supplementary Materials).

### 2.4. Determination of Antioxidant Activity

The determination of the antioxidant activity of extracts was performed by two different methods described by Alvaredo-López-Vizcaíno et al. [18]. The 2,2-azino-bis-3-ethylbenzothiazoline-6-sulphonic acid radical cation decolorization assay (ABTS) and the ferric reducing antioxidant power (FRAP), using Trolox as a standard. The analysis was performed in triplicate and results were reported as mg of Trolox equivalents/mg of dried *L. deliciosus* (mg TE/mg LD).

### 2.5. Cell Culture

The human cell line Caco-2 (clone TC7) was kindly provided by Dr. Edith Brot-Laroche (Université Pierre et Marie Curie-Paris 6, UMR S 872, Les Cordeliers, France). The cell line was maintained in a humidified atmosphere with 5% CO_2_ at 37 °C. Cells were cultured in Dulbecco’s modified Eagles medium (DMEM) (Gibco Invitrogen, Paisley, UK) supplemented with 20% fetal bovine serum (FBS) (Gibco Invitrogen, Paisley, UK), 1% non-essential amino acids (Gibco Invitrogen, Paisley, UK), 1% penicillin (1000 U/mL) (Gibco Invitrogen, Paisley, UK), 1% streptomycin (1000 μg/mL) (Gibco Invitrogen, Paisley, UK) and 1% amphotericin (250 U/mL) (Gibco Invitrogen, Paisley, UK). Cells were enzymatically treated with 0.25% trypsin-1 mM EDTA and sub-cultured in 25 cm^2^ plastic flasks at a density of 5 × 10^5^ cells/cm^2^. The culture medium was replaced every 2 days. Extract treatments were added 24 h after plating for assays in undifferentiated Caco-2 cells, and 15 days after plating on differentiated Caco-2 cells. Cell confluence (80%) was confirmed by optical microscopy observation [19,20]. LDE was diluted in a cell culture medium to a final concentration of 1.5 mg/mL, as determined by previous studies in our laboratory with other natural extracts.

### 2.6. Clonogenic Assay

A clonogenic assay was employed to ascertain the impact of LDE on the replicative immortality of colon cancer cells. Caco-2 cells were seeded in a TC-treated 96-well flat-bottom microplate (Corning^®^, Corning, NY, USA) at a density of 4 × 10^3^ cells/well and incubated for 24 h in an atmosphere with 5% CO_2_ at 37 °C. Subsequently, the medium was removed and replaced with a final volume of 100 μL of treatment medium containing 187, 250, 375, 500, 592.16, 750, and 1000 μg/mL of LDE, as described in the previous section. In each treatment, 1 × 10^3^ suspended cells in 500 μL of supplemented DMEM were seeded in triplicate in a TC-treated 24-well plate. The plates were then subjected to an incubation period of 14 days. Subsequently, the cells were fixed with buffered formalin for a period of 15 min. Following this, 0.5% crystal violet was applied and allowed to stain the cells for a duration of 45 min. The cells were then washed with H_2_O in order to remove the excess dye. Finally, images were captured. The colonies were enumerated, and the survival percentage was calculated as follows: the calculation of the percentage of colonies in treatments is performed by multiplying the number of colonies in treatments by 100 and dividing that result by the number of colonies in the control [21].

### 2.7. Adhesion Assay

To analyze changes in adhesion, Caco-2 cells were seeded in a TC-treated 24-well plate at a density of 2 × 10^4^ cells/well and incubated for 24 h in a 5% CO_2_ atmosphere at 37 °C. In the subsequent phase of the experiment, the cells were subjected to the treatment protocol outlined in Section 2.5. As previously indicated, cell cultures were maintained with varying concentrations of LDE for a period of 72 h. Following this period, both adhered and suspended cells were collected, stained with trypan blue (Sigma-Aldrich, Merck Millipore, Darmstadt, Germany), and counted. Subsequently, the suspended cells were reseeded in a TC-treated 24-well plate to analyze their adhesion. Following a 24 h period, both adhered and suspended cells were collected, stained with trypan blue, and counted. The images were captured using a specialized inverted microscope (AE31 EPI Motic, Hesse, Germany) that was integrated with a Nikon (model 111 camera, Hesse, Germany) and its accompanying 111 software (Hesse, Germany). The percentage of adhesion and dead cells in each treatment condition was calculated.

### 2.8. Cytotoxicity Determination

For the detection of cellular toxicity, cells were seeded in 96-well plates at a density of 4 × 10^3^ cells/well. The culture medium was substituted with a medium comprising LDE, and the cells were subjected to incubation for 48 or 72 h. The antiproliferative effect was gauged employing the Resazurin cell viability fluorometric assay [22], as modified by Quero et al. [23]. The effect on cell growth was expressed as a percentage of the control. The IC_50_ value was finally determined at 72 h of incubation. This value represents the concentration of the compound that reduces cell proliferation or viability by half. This value is employed in the ensuing analyses to evaluate the mechanism of action of LDE.

### 2.9. Cell Death Studies

In order to ascertain the type of LDE-induced cell death, cells were seeded in 25 cm^2^ flasks (5 × 10^5^ cells/cm^2^) with the extracts at the IC_50_ concentration for 48 h. Thereafter, the cells were harvested and stained with Annexin V-FITC and propidium iodide, as previously described [24]. A negative control of untreated cells was prepared to define the basal level of apoptotic and necrotic or dead cells. Following the incubation period, the cells were transferred to flow cytometry tubes and analyzed by flow cytometry (Beckman Coulter, Brea, CA, USA). The subsequent methodology was employed in accordance with the approach previously delineated by Quero et al. [23].

### 2.10. Analysis of Cell Cycle and DNA Content

The cells were initially seeded in 25 cm^2^ flasks. Following treatment, the cells were fixed in 70% ice-cold ethanol, subsequently stored at 4 °C for a period of 24 h, and then subjected to centrifugation. Thereafter, the cells were rehydrated in PBS and stained with a solution containing both propidium iodide [PI; final concentration, 50 μg/mL] (Sigma-Aldrich, Merck Millipore, Darmstadt, Germany) and RNase A [100 μg/mL] (Sigma-Aldrich, Merck Millipore, Darmstadt, Germany). The analysis of PI-stained cells was conducted to determine the DNA content, utilizing a FACS ARRAY BD (BD Biosciences, Franklin Lakes, NJ, USA) equipped with an argon ion laser. The red fluorescence emitted by PI was collected using a 620 nm longer pass filter as a measure of the amount of DNA-bound PI and displayed on a linear scale. The distribution of cell cycles was determined on a linear scale. The percentage of cells in cycle phases was determined using MODIFIT 3.0 verity software (Washington, DC, USA)

### 2.11. The Determination of Mitochondrial Membrane Potential and Caspase-3 Activity by Flow Cytometry

In order to ascertain the mitochondrial potential and the activity of caspase-3, the cells were initially seeded in 25 cm^2^ flasks. Thereafter, the cells were exposed to LDE for a period of 48 h. The control cells were incubated with a medium devoid of any extracts. Subsequently, the aforementioned methodology, previously delineated by this research group, was employed [23].

### 2.12. Determination of Intracellular Levels of Reactive Oxygen Species (ROS)

Cells were seeded in 96-well plates at a density of 4 × 10^3^ cells/well. The intracellular level of ROS was assessed using the dichlorofluorescein assay, as previously described [20,23]. Cells were cultured for 24 h before being incubated with LDE for 48 h. Subsequently, the medium was removed, and the cells were washed twice with PBS and incubated for 1 h with 20 µM of 2′,7′-dichlorofluorescein diacetate (DCFH-DA) (Sigma-Aldrich, Merck Millipore, Darmstadt, Germany) in PBS at 37 °C. The formation of the fluorescence-oxidized derivative of DCF was monitored at an emission wavelength of 535 nm using an excitation of 485 nm on a FLUOstar Omega multiplate reader (BMG Labtech, Ortenberg, Germany). ROS levels were measured by assessing the fluorescence at time “zero” and after 20 min of incubation at 37 °C. Fluorescence intensity values were expressed as a percentage compared to the control, reflecting the total ROS content. The same procedure was applied to the differentiated Caco-2 cells after they reached confluence.

### 2.13. mRNA Expression Levels of NOS2, PTGS2, IL-6 and IL-8

In order to investigate the anti-inflammatory effect of LDE in undifferentiated Caco-2 cells, the cells were seeded in a 6-well plate at a cell density of 5.7 × 10^4^ cells/cm^2^. Then, the cells were treated with LDE at 490 μg/mL (IC_50_) for 24 h. Following the treatment period, the cells were subjected to evaluation of the pro-inflammatory genes *NOS2*, *PTGS2*, *IL-6* and *IL-8* by quantitative reverse transcription PCR (RT-qPCR). RNA extraction was performed using Quick-RNA Miniprep Kit (ZYMOresearch, Frieburg, Germany), The quality, purity and concentration of the RNA were validated using an agarose gel electrophoresis and a LVIS plate in SPECTROstar Nano plate reader. Subsequently, 500 ng of total RNA were converted into complementary DNA (cDNA) utilizing the PrimeScript™ RT Master Mix kit (TaKaRa Biotechnology, Kusatsu, Shiga, Japan). Changes in mRNA expression were determined by quantitative RT-PCR (RT-qPCR). cDNA synthesis was performed using the First Strand synthesis kit (Thermo Scientific, Madrid, Spain). SYBR Green PCR Master Mix (Applied Biosystems, Foster City, CA, USA) was used to analyze gene expression by qPCR. Specific primers, designed and checked as previously described, were purchased from Applied Biosystems. Sequences are shown in Supplemental Table S2. The analysis of the data was conducted using the comparative ΔCt method, with results expressed as 2^−ΔΔCt^ after normalizing gene expression to the endogenous control *GAPDH*. The results were expressed as a relative change in gene expression between the control sample and the treated sample.

### 2.14. Statistical Analysis

All extractions were conducted using three independent batches of *L. deliciosus*. Each extract was analysed in at least triplicate (analytical replicates), and all biological assays were carried out with a minimum of three independent replicates. The data are expressed as the mean ± standard deviation. The means were compared using one-way analysis of variance (ANOVA). To ascertain significant differences, a Bonferroni or Tukey multiple comparison test was employed. Statistical analysis and graphing were performed using GraphPad Prism Version 5.02 software on a personal computer (GraphPad, San Diego, CA, USA).

## 3. Results and Discussion

Apart from their nutritional value, mushrooms are notable for their high content of bioactive compounds, which suggests potential applications in therapeutic contexts. *L. deliciosus* is a prevalent and well-regarded edible mushroom, esteemed for its gustatory qualities, flavor, and textural characteristics [8]. A wide range of biological activities of *L. deliciosus* has been demonstrated in several studies, including antioxidant, anti-inflammatory, antimicrobial, antihyperglycemic, immunomodulatory, antiproliferative, and anticancer activity [5,25,26,27].

Moreover, research evaluating the impact of *L. deliciosus* on intestinal cancer remains limited. Therefore, the objective of the present study was to assess the potential and mechanism of action of LDE in Caco-2 cells. The potential impact of these extracts on cell adhesion and metastasis was also assessed. In addition, the potential antioxidant and anti-inflammatory properties of the LDE were investigated.

### 3.1. Extraction of LDE

The selection of the NaDES formulations used in this study was grounded in extensive evidence demonstrating their suitability for the extraction of phenolic compounds. Several studies have shown that NaDES based on glycerol, amino acids, sugars, and choline chloride possess strong hydrogen-bonding networks, tunable polarity and enhanced solubilisation capacity for phenolic molecules. For example, Mansinhos et al. reported that glycerol-based and choline-based NaDES significantly improved the extraction of phenolic compounds from *Lavandula pedunculata*, particularly when combined with green extraction techniques [16]. Ferreira-Santos et al. further highlighted the advantages of these NaDES families, including their biodegradability, biocompatibility, and high affinity for polyphenols, in the context of emerging green extraction strategies [15]. Their conclusions are consistent with the work of Benítez-Correa et al., who demonstrated that NaDES composed of choline chloride with polyols (e.g., glycerol or ethylene glycol) efficiently extracted phenolic acids, flavonoids and other bioactive phytochemicals from plant materials [28]. These studies collectively support the suitability of the NaDES selected in this work: glycerol/glycine/water (7:1:3), glycerol/glucose (2:1), choline chloride/ethylene glycol (1:2) and choline chloride/glycerol (1:2).

The molar ratios used in these NaDES systems were chosen according to established eutectic formation principles and previous reports identifying optimal hydrogen-bond acceptor (HBA)–hydrogen-bond donor (HBD) combinations for phenolic extraction. Ratios with an excess of HBD (e.g., 1:2 for choline chloride/polyol systems) are known to effectively depress the melting point of the mixture, reduce viscosity and provide the polarity range required for dissolving phenolic molecules. These ratios correspond to those most frequently reported in the DES literature for efficient polyol-based NaDES [28]. The 7:1:3 ratio in the glycerol/glycine/water NaDES was selected to maximise the density of hydrogen-bonding sites: glycerol as a multi-OH HBD, glycine as a zwitterionic stabiliser and water as a viscosity-modifying additive, while maintaining a stable eutectic structure, a rationale supported by Mansinhos et al. [16]. Likewise, the 2:1 glycerol/glucose formulation provides a highly hydrophilic, sugar-rich solvent capable of interacting strongly with phenolic hydroxyl groups, consistent with the suitability of sugar-based NaDES highlighted in recent reviews [15].

Taken together, the selected NaDES and their molecular ratios reflect compositions repeatedly validated in the literature for their ability to enhance the extraction, stabilization and solubilization of phenolic compounds, while respecting green-chemistry criteria.

**Figure 1:**
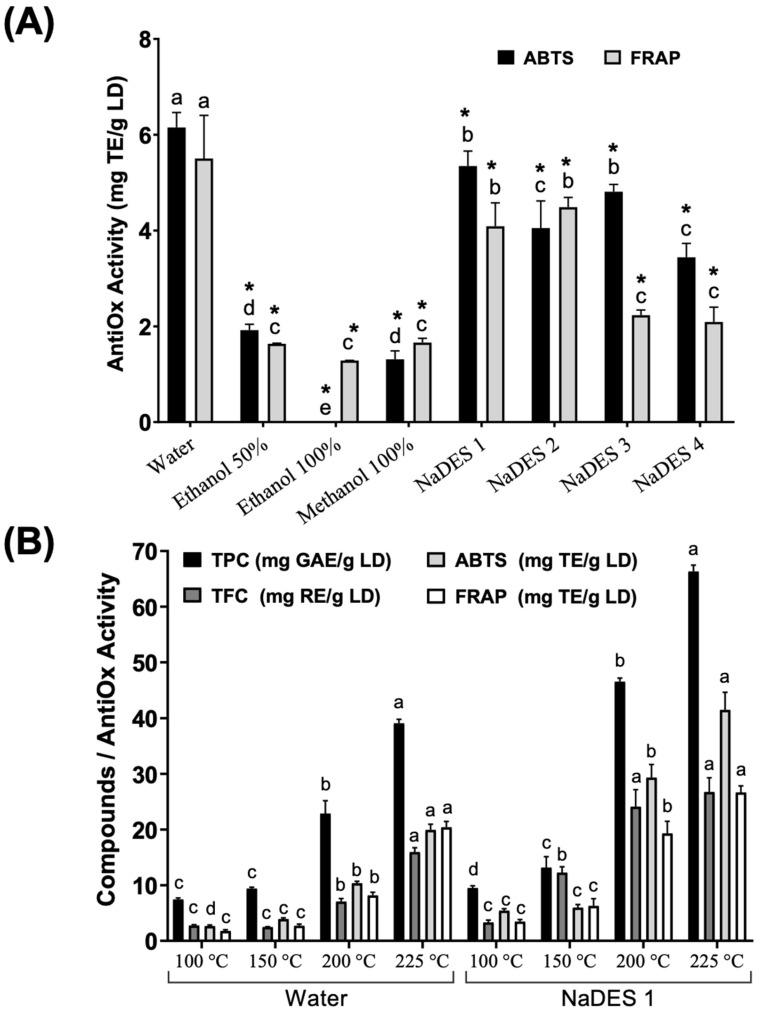
Total phenolic content (TPC), total flavonoid content (TFC) and antioxidant activity (ABTS and FRAP) of *L. deliciosus* obtained by (**A**) different solvents, and (**B**) microwave-assisted extraction (MAE) using selected solvents (Water and NaDES 1) at different temperatures (100, 150, 200 and 225 °C). Bars represent the mean ± standard error (n = 3 or 4). Different letters indicate significant differences separately for each method according to Tukey’s test (α = 0.05); * marks solvents that are significantly different from the best-performing solvent (water) within the same method.

In our study, the selection of the most suitable extraction solvents was guided by the antioxidant activity of LDE, which was assessed through the ABTS and FRAP assays. These assays serve as reliable indicators of the presence of active compounds, such as phenolic compounds, which contribute to the antioxidant potential of the extracts. This renders them valuable for food, nutraceutical, and therapeutic purposes. The results of this study are illustrated in Figure 1A. Water and several NaDES, particularly NaDES 1 composed of glycerol/glycine/water in a 7:1:3 molar ratio and diluted with 30% water, exhibited the highest antioxidant capabilities, whereas extracts based on ethanol and methanol displayed markedly lower values. The findings indicate that solvent polarity and composition have a significant impact on the recovery of antioxidant biomolecules.

As mentioned before, from a green chemistry perspective, water and NaDES solvents are regarded as environmentally friendly, non-toxic, and biodegradable, making them suitable substitutes for traditional organic solvents in food, cosmetic, and pharmaceutical uses [29,30]. In accordance with the investigations reported in the extant literature [15,16], the present study demonstrates that water and NaDES 1 are the most effective, safe, and sustainable solvents. These solvents were therefore selected for further comparison under different extraction conditions.

MAE using both solvents was performed at temperatures between 100 and 225 °C. As illustrated in Figure 1B, elevated temperatures led to a substantial enhancement in the extraction yields of total phenolic content (TPC), along with an augmentation in antioxidant activity, as determined by ABTS and FRAP assays. The enhancement in extraction efficacy with rising temperature can be ascribed to many physicochemical phenomena of MAE, such as cell wall disintegration, diminished solvent viscosity, augmented solute transport, and improved solubility of phenolic compounds. Comparable patterns have been observed in the MAE of plant and fungal matrices, wherein elevated temperatures and microwave powers have been shown to enhance phenolic compounds extraction and antioxidant efficacy [17,31].

In the present study, the two solvents evaluated were subjected to a rigorous testing process, with the objective of ascertaining the most efficacious option for the purpose of extraction. The results obtained revealed that NaDES 1 consistently yielded superior extraction results and antioxidant activity in comparison to water across the entire range of temperatures. This distinction was particularly evident at temperatures of 200 and 225 °C. The exceptional performance of NaDES can be attributed to its robust hydrogen-bond network and adjustable polarity, which enhance the solvation and stabilization of phenolic and flavonoid compounds during thermal processing [30]. The concurrent increase in TPC, TFC, and antioxidant activity suggests a robust correlation, indicating that phenolic and flavonoid molecules are the predominant contributors to the antioxidant potential of LDE.

In conclusion, NaDES 1, composed of a 7:1:3 molar ratio of glycerol/glycine/water and diluted with 30% water, at a temperature of 225 °C under MAE, yielded the highest concentration of antioxidant phenolic compounds. This outcome emphasizes its potential as a non-toxic and eco-friendly solvent for generating antioxidant-rich extracts from mushrooms, specifically *L. deliciosus*.

### 3.2. Chemical Composition of LDE

At present, phenolic compounds have garnered significant interest from the scientific community due to their potential application as prophylactic, antioxidant, and therapeutic agents in a variety of diseases [32]. These compounds function as free radical scavengers, thereby providing protection against a wide range of diseases such as cancer, diabetes, cardiovascular diseases, neurodegenerative diseases, autoimmune disorders, and certain inflammatory diseases [33,34]. Phenolic compounds represent a prominent class of plant-based anticancer drugs, owing to their noteworthy efficacy, whether as monotherapy or in combination with other anticancer agents [35].

The individual phenolic composition of LDE, produced using water and NaDES 1, was determined via HPLC-ESI-MS (Table 2). A total of five principal phenolic acids were identified in the sample: p-coumaric, 3,4-dihydroxybenzoic, gallic, 4-hydroxybenzoic, and vanillic acids. In addition to the phenolic acids identified, HPLC-MS analysis revealed the presence of a flavonoid, rutin, in low concentrations (64–87 µg/100 g of dry LDE).

The NaDES 1 extracts exhibited considerably larger quantities of all detected phenolic compounds than the water extracts. This finding validates the superior extraction efficiency of the NaDES system. Vanillic acid was the predominant constituent in both extracts, with concentrations of 2590 µg/L in NaDES 1 (28,781 µg/100 g of dry extract), which is approximately 111 times greater than in the water extract. The other phenolic compounds that were found to be prevalent included 4-hydroxybenzoic acid and gallic acid. These two phenolic compounds exhibited enhancements ranging from two- to threefold in NaDES 1 compared to water. The enhanced solubilization of these compounds can be attributed to the robust hydrogen-bond structure and polarity adjustment of NaDES 1 (glycerol/glycine/water, 7:1:3 mol/mol + 30% water), which facilitates the dissolution and stabilization of hydrophilic phenolic compounds during extraction.

According to the extant literature on *L. deliciosus* and other *Lactarius* species, gallic, vanillic, p-coumaric, and 4-hydroxybenzoic acids have been identified as significant phenolic components that enhance the antioxidant capacity of their extracts [36]. The findings indicated that *L. deliciosus* had moderate levels of hydroxybenzoic and hydroxycinnamic acids, aligning with the phenolic profile identified in our work. The prevalence of vanillic and gallic acids is consistent with their common identification as principal phenolic compounds in wild edible mushrooms, where they significantly enhance antioxidant properties [37]. The enhanced extraction efficiency achieved with NaDES 1 is consistent with existing literature that suggests NaDES enhances the recovery of phenolic acids relative to traditional solvents [15]. Furthermore, the prevalence of phenolic acids, including vanillic, gallic, and 4-hydroxybenzoic acids, in the extracts elucidates the elevated antioxidant activity quantified by ABTS and FRAP assays (Figure 1B), as these compounds are recognized for their potent radical-scavenging and redox capabilities [16]. In addition, plant-based polyphenolic compounds have found application in chemotherapy and radiotherapy for cancer treatment, as they exhibit negligible side effects on normal cells and other organs [38]. Polyphenolic compounds have demonstrated the capacity to combat cancer cells, as evidenced by their ability to induce the expression of p21, p53, and p27 genes. These genes, in turn, can trigger cell cycle arrest in the G1, S, and G2 phases [39].

### 3.3. Effect of LDE on Caco-2 Cell Viability

Nowakowski et al. have conducted studies in which they have determined that the possible antioxidant action of phenolic compounds and other compounds, such as sesquiterpenes from the ethanolic extract of *L. deliciosus*, has effects on the cell cycle, specifically in the sub-G1 phase. They have also determined that there is an inhibitory effect on the activity of metalloproteinases 2 and 9 at a concentration of 500 μg/mL on the glioblastoma cell line LN-18. These effects have been shown to be superior compared to the effects of aqueous and hydroalcoholic extracts and extracts from other edible mushrooms [8]. In this study, we examined the effects of our extracts, which were composed primarily of phenolic compounds, including 4-hydroxybenzoic and vanillic acids, on the proliferation of Caco-2 cells. The assays were conducted at the following concentrations: 250, 375, 500, 750, and 1000 µg/mL. The assays were also conducted at two incubation times: 48 and 72 h.

**Figure 2:**
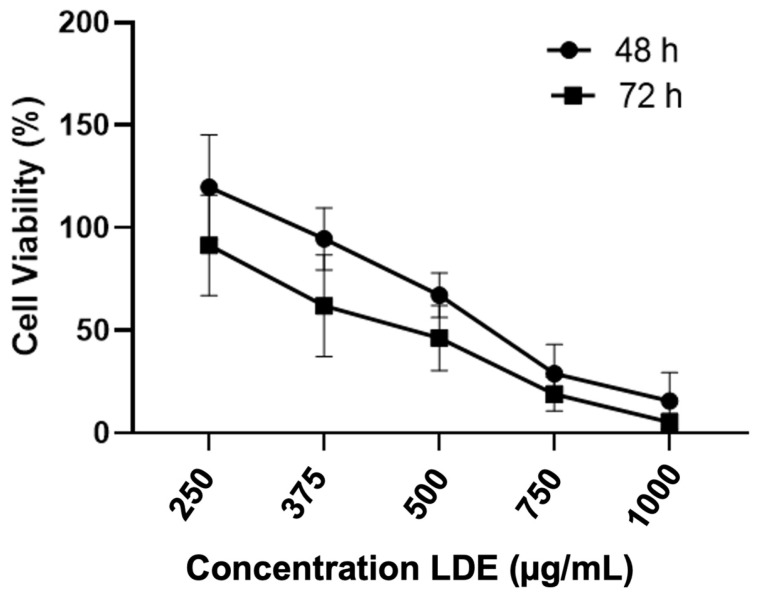
The antiproliferative effect of LDE, at varying concentrations, on Caco-2 cells was evaluated following an incubation period of 48 or 72 h, using three replicates.

As demonstrated in Figure 2, LDE exhibited a dose- and time-dependent decline in the viability of Caco-2 cells. According to these results, LDE exhibited a significant antiproliferative effect. The findings demonstrated that an IC_50_ value of 592.16 µg/mL was attained after 48 h of exposure to LDE, while a value of 490 µg/mL was achieved following 72 h of exposure. Furthermore, the NaDES 1 solvent without extract did not demonstrate any cytotoxic effects on cells. For subsequent experiments, the IC_50_ concentration (490 µg/mL) corresponding to 72 h has been selected. The incubation time that was subsequently employed in the ensuing assays was 48 h, in order to facilitate a meaningful comparison with the extant literature. This comparison was made with regard to the incubation time employed in previous studies carried out in our laboratory, which involved another mushroom, such as *Boletus edulis* [40].

### 3.4. Clonogenic Survival of Caco-2 Cells

**Figure 3:**
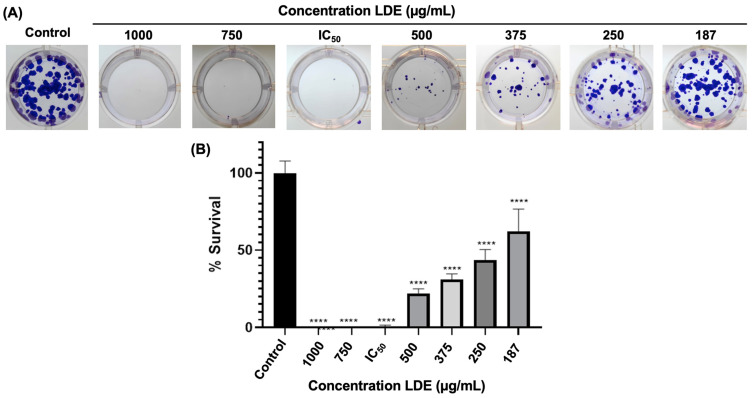
Clonogenic assay of LDE. (**A**) Number of colonies formed, stained with 0.5% crystal violet, after treatments with LDE or DMEM medium (Control) for 48 h, followed by 14 days of incubation. (**B**) Quantification of colonies. **** *p* ≤ 0.0001 vs. control. IC_50_ = 592.16 μg/mL.

Following 48 h exposure to LDE, Caco-2 cells demonstrated signs of recovery and were subsequently reseeded at a low density to assess the impact of the extract on their clonogenic survival. The capacity of cells to proliferate and form colonies was influenced by the treatment in a dose-dependent manner (Figure 3A). The calculation of the survival percentage indicated that treatment with LDE at concentrations of 187, 250, 375, and 500 μg/mL resulted in 62.14%, 43.50%, 31.07%, and 22.03%, respectively. The calculated IC_50_ value indicates a nearly complete decrease of 0.56% in the number of colonies formed after treatment. In summary, the administration of LDE resulted in a 100% reduction in colony formation at the calculated IC_50_ concentration (Figure 3B). The quantification of colonies demonstrated a decrease in clonogenic survival as the extract concentration in the medium increased. At a concentration of 250 μg/mL, the survival decreased nearly by half, and at the calculated IC_50_ concentration, it approached zero.

It has been documented that certain isolated polysaccharides have the capacity to stimulate a cell-mediated immune response, thereby engendering an antitumor effect [41]. The probable existence of these compounds in the LDE is in accordance with prior reports [7]. In consideration of the results obtained in this study, it is hypothesized that the combination of the extract, which is characterized by a high content of diverse compounds, including polysaccharides, phenolic compounds, and sesquiterpenes, exerts a specific effect on the Caco-2 cell line. This phenomenon may involve alterations to the cytoskeleton, potentially leading to a reduction in cell adhesion and exerting a concurrent influence on clonogenic survival.

### 3.5. LDE Effects on Cell Adhesion of Caco-2

**Figure 4:**
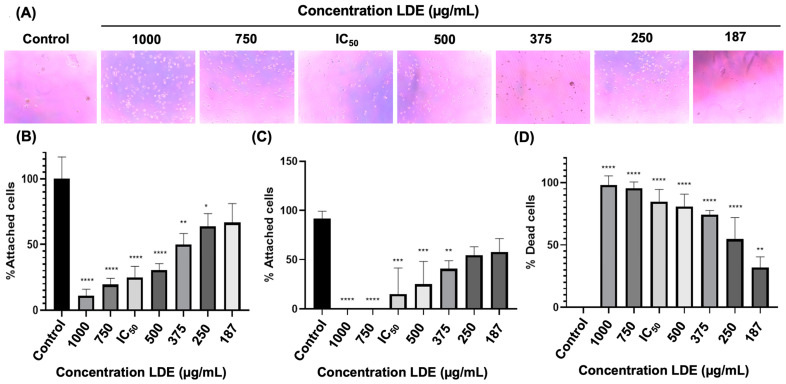
Adhesion cell assay of LDE. (**A**) Images illustrating the adhesion capacity of cells from the medium recovered from each well after LDE treatment and reseeded after 48 h (Magnification 10×). (**B**) Percentage of cells adhered to the monolayer following treatment with LDE for a period of 48 h. (**C**) Percentage of adhesion of previously treated suspended cells that were recovered and reseeded after 48 h with LDE. (**D**) Analysis of cell death in the population of non-adherent cells. * *p* ≤ 0.05; ** *p* ≤ 0.01; *** *p* ≤ 0.001; **** *p* ≤ 0.0001 vs. control. IC_50_ = 592.16 μg/mL.

The alterations in cell adhesion capacity were evaluated after 48 h treatments. The results are presented in Figure 4B. Treatment of cells with LDE at the lowest concentration used, 187 μg/mL, resulted in a significant compromise in cell adhesion, with a value of 66.66% (*p* = 0.0543) compared to the control group. These findings suggest that the capacity of the cells was affected even at concentrations below the calculated IC_50_. Furthermore, an increase in extract concentration resulted in a concomitant decrease in adhesion percentage, with statistically significant differences observed in comparison to the control group. At a concentration of 250 μg/mL, 63.88% of cells remained adhered to the surface (*p* = 0.0073) and at 375 μg/mL results 50% (*p* = 0.0003) of adhered cells. A concentration of LDE higher than 500 μg/mL results in a percentage of adhered cells lower than 50% (500 μg/mL = 30.55%, for 592.16 μg/mL (i.e., calculated IC_50_) = 25%, 750 μg/mL = 19.44%, and 1000 μg/mL = 8.33%).

In a previous study, it was observed that cells that detached during normal cell line maintenance retained their adhesion capacity. Given its potential relevance to the metastatic behavior of metastatic cancer, we sought to determine if cells detached after 48 h of treatment maintained this capacity. The adhesion assessment revealed that cells detached after treatment with a concentration of 187 and 250 μg/mL of the extract at 48 h. The adhesion capacity of the cells was 57.79% (*p* = 0.1250) and 54.50% (*p* = 0.0753), respectively. The control group showed a percentage of 91.90% (Figure 4A,C). In contrast, substrate binding was found to be influenced by treatments with 375, 500, 750, and 1000 μg/mL, as well as the calculated IC_50_, with statistical differences observed in comparison to the control. Following incubation with a concentration of 375, only 40.66% of cells exhibited adhesion (*p* = 0.0076). At a concentration of 500 μg/mL, the adhesion rate was 24.95% (*p* = 0.0006). At a concentration of 750 μg/mL, no adhesion was observed (*p* < 0.0001). Similarly, at a concentration of 1000 μg/mL, no adhesion to the substrate was detected (*p* < 0.0001). Finally, cell death in this population of non-adherent cells was analyzed. For control cells, a value of 0% positive cells for trypan blue staining was obtained, while at 187 μg/mL it was 31.92% (*p* = 0.01). The percentages of cell death in cells treated with 250, 375, 500, IC_50_, 750, and 1000 μg/mL in the medium were statistically different from the control (*p* < 0.0001), with 54.66%, 74.19%, 80.76%, 84.75%, 95.59%, and 98.11%, respectively (Figure 4D).

### 3.6. Induction of Cellular Death (Apoptosis, Cell Cycle, Mitochondrial Membrane Potential and Caspase 3 Activity) by LDE

The primary pathway for regulating tissue development and homeostasis is apoptosis. Maintaining a state of balance between tissue proliferation and cell death is imperative for preserving the natural state of cells [19,20]. To elucidate the mechanism by which our LDE inhibited Caco-2 cell proliferation, the type of cell death produced was studied. Apoptosis is a type of programmed cell death that can be triggered by different signals in target cells. The culmination of these signals results in a singular event: the loss of mitochondrial membrane permeability, which subsequently leads to the release of cytochrome c into the cytosol. This process initiates a cascade of events, including caspase activation, membrane blebbing, DNA fragmentation, and ultimately, cell death [42].

**Figure 5:**
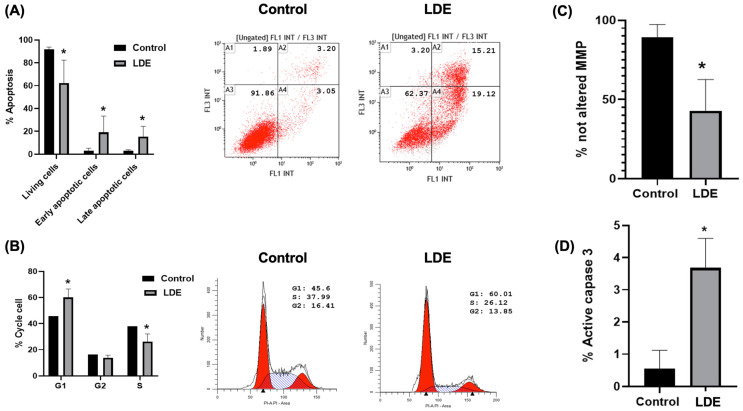
Caco-2 cells incubated for 48 h under control or treated with LDE (490 µg/mL; IC_50_). (**A**) Apoptosis analysis by flow cytometry. (**B**) Cell cycle analysis by flow cytometry. (**C**) Mitochondrial membrane potential (MMP) assay. (**D**) Caspase 3 activity. * *p* ≤ 0.05 vs. control.

Given the presence of phenolic compounds in the LDE, and the previous demonstration of these compounds’ ability to induce apoptotic cell death [43], it was deemed worthwhile to investigate this particular form of cell death. The results demonstrated that LDE induced apoptosis (Figure 5A). Mitochondrial depolarization is one of the initial steps that lead to apoptosis, and the process of apoptosis is executed by the activation of caspase-3. Consequently, the potential alterations in mitochondrial membrane potential and caspase-3 activity were subjected to rigorous scrutiny. The results demonstrated that LDE significantly altered the number of cells with mitochondrial membrane potential changes (Figure 5C) and activated caspase-3 (Figure 5D). Consequently, these findings provide a rationale for the apoptosis observed in the cells (Figure 5A).

A number of studies have been conducted on LS180 cells, a type of human colon adenocarcinoma, using a polysaccharide-rich fraction of *Cantharellus cibarius*. These studies have shown that treatment with this fraction results in an increase in the percentage of cells in the G1 phase of the cell cycle [44]. In this manner, the influence of LDE on cell cycle progression was examined using flow cytometry. The results of the study demonstrated that the administration of this extract resulted in the accumulation of Caco-2 cells in the G0/G1 phase of the cell cycle, accompanied by a concomitant decrease in cancer cells in the S phase (Figure 5B). These findings are consistent with those previously reported by our research group in Caco-2 cells treated with the *Boletus edulis* mushrooms [40].

### 3.7. Effect of LDE on Intracellular ROS Levels

**Figure 6:**
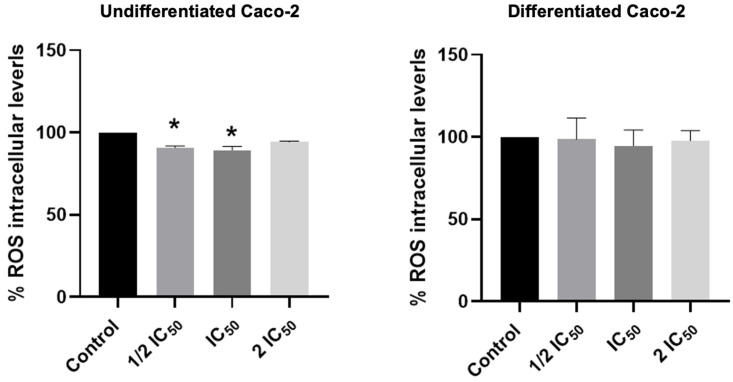
Measurements of intracellular ROS levels after 48 h incubation with LDE at 245, 490 and 980 µg/mL (1/2 IC_50_, IC_50_, and 2 IC_50_, respectively). * *p* ≤ 0.05 vs. control cells.

The mechanism of action of the LDE may be related to its antioxidant activity. The generation of reactive oxygen species (ROS) and oxidative stress has been demonstrated to contribute to the development of numerous disorders, including tissue damage, cardiovascular diseases, kidney diseases, liver diseases, neurodegenerative diseases, and cancer [45]. These phenomena can be attributed to the activation of signaling pathways or mitochondrial dysfunction [46]. The antioxidant properties of phenolic compounds found in plants and mushrooms are attributed to their hydroxyl groups, which act as effective electron donors [47]. The LDE demonstrated cellular antioxidant activity at concentrations of 245 and 490 µg/mL, as substantiated by the radical scavenging assay. This assay is capable of neutralizing reactive species, such as ROS, which are known to induce cellular damage, cell death, and cancer development (Figure 6).

It is imperative to acknowledge the pivotal function of bioactive compounds derived from plants and fungi in the prevention of gastrointestinal diseases, a consequence of the impact of free radicals. Given their high antioxidant capacity and content of bioactive ingredients, and in consideration of the findings of the antioxidant effect determined by FRAP and ABTS (Figure 1 and Table 2), it was deemed worthwhile to evaluate the effects of the LDE at varying concentrations and after 48 h of incubation in an intestinal barrier model (differentiated cells). This cell line has been observed to spontaneously acquire the phenotypic characteristics of noncancerous enterocytes after reaching confluence. Caco-2 monolayer cells form tight junctions and exhibit the polarized columnar morphology of enterocytes, expressing functional microvilli on the apical membrane [48,49,50,51]. Consequently, differentiated Caco-2 cells have been established as an acceptable in vitro intestinal barrier model. The results demonstrated that the LDE exerted no effect on the cells, as they were not subjected to stress (Figure 6). Consequently, the LDEs are devoid of any adverse effects. The antioxidant capacity of plant extracts exhibits a strong correlation with their clinical application in gastrointestinal diseases associated with oxidative stress [52,53]. The results obtained with LDE suggest that they could have potential application in the management of gastrointestinal diseases related to oxidative stress, such as cancer and inflammatory bowel diseases [5,7,8,10].

### 3.8. Anti-Inflammatory Effect of LDE

**Figure 7:**
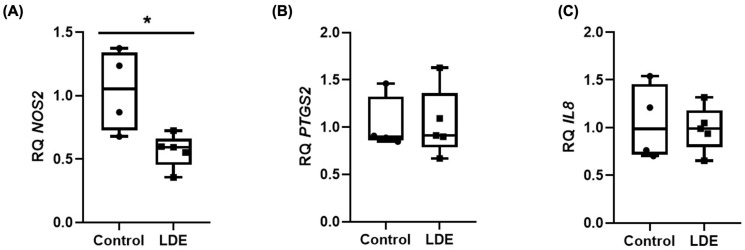
Gene expression of (**A**) *NOS2*, (**B**) *PTGS2* and (**C**) *IL-8* by RT-qPCR in Caco-2 cells. Cells were treated with 490 μg/mL of LDE or not (control cells) for 24 h. The level of the target mRNA was normalized to the level of *GADPH* and expressed as a relative quantification (RQ) in which control expression was arbitrarily set to 1. Data are expressed as boxes (means ± SD) and whiskers (min to max). (* *p* ≤ 0.05).

A multitude of studies have demonstrated the bioactive properties of mushroom extracts and their secondary metabolites. These properties include antioxidant, antitumor, antimicrobial, and anti-inflammatory activities, among others [54]. The anti-inflammatory activity of the subject has been associated with a reduction in the production of nitric oxide (NO) and other inflammatory mediators, including interleukins (IL-1β, IL-6, IL-8), tumor necrosis factor (TNF-α), and prostaglandin E2 (PGE2). This ultimately leads to a decrease in inflammation [25,55,56,57,58,59,60]. The findings of this study demonstrate that treatment of Caco-2 cells with 490 μg/mL of LDE for 24 h resulted in a 50% reduction in the expression of *NOS2* in comparison with the control cells, while the expression levels of *PTGS2* and *IL-8* remained comparable to those of the control group (Figure 7), and no *IL6* expression was detected in any of the explored conditions. These results underscore the robust anti-inflammatory potential of LDE in colon cancer cells, particularly through the downregulation of *NOS2*. These findings are consistent with those previously obtained in our group, in which a treatment with 1500 μg/mL of *Boletus edulis* extracts for 48 h resulted in a reduction in the expression of *NOS2* and *PTGS2* in undifferentiated Caco-2 cells [40]. In a similar vein, Moro et al. demonstrated that 500 μg/mL of LDE was able to downregulate inflammatory signaling in LPS-stimulated RAW 264.7 macrophages, achieving this by reducing both NO production and the mRNA expression of *NOS2*, *IL-1β*, and *IL-6* [25]. Concurrently, Han et al. evaluated the anti-inflammatory effect of *Cordyceps militaris*, a well-known medicinal mushroom, and found that these extracts reduced inflammation by down-regulating *NOS2* and *TNF-α* mRNA expression in both DSS-induced colitis and in LPS-stimulated RAW264.7 cells [61].

## 4. Conclusions

The findings of the present study indicate that solvent polarity and composition have a significant impact on the recovery of antioxidant biomolecules. The highest concentration of antioxidant phenolic compounds was obtained using the NaDES glycerol/glycine/water-assisted by MAE at a temperature of 225 °C. LDE has been found to contain a significant quantity of phenolic compounds, with 4-hydroxybenzoic acid and vanillic acid being the predominant ones. The presence of these compounds in the extract suggests their potential to act as antioxidants, which may indicate their efficacy as anticancer agents. The present study showed that the treatment with LDE significantly reduced colony formation and cell adhesion in a dose-dependent manner, leading to nearly complete inhibition of clonogenic survival at the IC_50_ concentration and a marked increase in cell death among non-adherent colon cancer cells. In addition, this work demonstrates that LDE exerts its anti-proliferative effects on human colon carcinoma cells (Caco-2) by inducing cell cycle arrest in the G0/G1 phase and promoting apoptosis, accompanied by alterations in mitochondrial potential and an augmentation in caspase-3 activity. The LDE exhibited an antioxidant effect, as evidenced by a decrease in ROS levels within cancer cells. In addition, these extracts demonstrated an anti-inflammatory effect by a substantial decrease in *NOS2* gene expression. While these results highlight the promising biological activities of LDE, the study acknowledges a few limitations. Specifically, the extraction conditions examined represent a comparison of selected parameters rather than a comprehensive optimization procedure. Therefore, a complementary and systematic optimization study is needed to more precisely determine optimal extraction conditions. Moreover, future research should also explore the in vivo effects of LDE to validate its potential as a therapeutic or functional ingredient.

## Figures and Tables

**Table 1:** The formulations of the selected NaDES for extracting the targeted bioactive compounds.

Name	Formulation	Molecular Ratio	Water (% *v*/*v*)
NaDES 1	Glycerol/glycine/water	7:1:3	30
NaDES 2	Glycerol/glucose	2:1	30
NaDES 3	Choline chloride/ethylene glycol	1:2	30
NaDES 4	Choline chloride/glycerol	1:2	30

**Table 2:** Concentration of individual phenolic acids identified in LDE obtained using water and NaDES 1 (glycerol/glycine/water). Data are expressed as µg/L of extract and µg/100 g of dry extract (mean ± SD, n = 3).

Phenolic Compound	Water	NaDES 1	Water	NaDES 1
	µg/L (LDE Liquid)		µg/100 g of Dry LDE	
*p*-Coumaric acid	0.25 ± 0.04	7.2 ± 0.5	2.7 ± 0.4	80.9 ± 6.1
3,4-Dihydroxibenzoic acid	5.07 ± 0.08	26.6 ± 2.3	56.3 ± 0.8	295.8 ± 26.3
Gallic acid	20.0 ± 0.2	36.3 ± 3.5	222.0 ± 2.3	403.6 ± 39.1
4-Hydroxibenzoic acid	28.6 ± 0.7	83.0 ± 7.2	318.0 ± 8.5	922.0 ± 80.2
Vanillic acid	23.2 ± 1.2	2590.3 ± 23.3	258.3 ± 14.2	28,781 ± 259
Rutin	5.8 ± 0.01	7.8 ± 0.6	64.1 ± 0.1	87.1 ± 6.2

LDE: *Lactarius deliciosus* extract.

## Data Availability

Data are contained within the article and Supplementary File.

## Supplementary Materials

The following supporting information can be downloaded at: https://www.mdpi.com/article/10.3390/antiox14121452/s1, Table S1: Retention time (RT), mass spectra (*m*/*z* of the ions), and calibration curves of identified phenolic compounds; Table S2: Characteristics of primers used in RT-qPCR according to MIQE guidelines.
