# Supplementary material for: Lactarius deliciosus Extract from Green Microwave-Assisted Eutectic Solvent Extraction as a Therapeutic Candidate Against Colon Cancer
Source: Antioxidants (Basel). 2025 Dec 2;14(12):1452. doi: 10.3390/antiox14121452 (PMC12730110; doi:10.3390/antiox14121452)
Supplement: Supplementary file 1 [file antioxidants-14-01452-s001.zip › antioxidants-3983060-supplementary.pdf]

Supplementary file

Lactarius deliciosus Extract from Green Microwave-Assisted Eutectic Solvent Extraction as a Therapeutic Candidate Against Colon Cancer

Seyed Hesamoddin Bidooki [1], Beatriz Rodríguez-Martínez [2,3], Javier Quero [4], Luis Vicente Herrera-Marcos [4], Mónica Paesa [5,6,7], Marina Delgado-Machuca [8], Oscar F. Beas-Guzmán [8], Jesús Osada [1,9,10], Pedro Ferreira-Santos [2,3,*] and María Jesús Rodríguez-Yoldi [4,9,10,*]

[1] Department of Biochemistry and Molecular and Cellular Biology, Faculty of Veterinary Medicine, Health Research Institute of Aragon-University of Zaragoza, 50013 Zaragoza, Spain; S.H.B (sbidooki@unizar.es), J.O (josada@unizar.es)

[2] Department of Chemical Engineering, Faculty of Science, University of Vigo (Campus Ourense), As Lagoas, 32004 Ourense, Spain.; B.R.M (beatriz.rodriguez@uvigo.es), P.F.S (pedromiguel.ferreira@uvigo.gal)

[3] Instituto de Agroecoloxía e Alimentación (IAA). University of Vigo (Campus Auga), As Lagoas, 32004 Ourense, Spain.

[4] Department of Pharmacology and Physiology, Legal and Forensic Medicine, Faculty of Veterinary Medicine, Aragon Health Research Institute, University of Zaragoza, 50013 Zaragoza, Spain; J.Q (javierquero94@gmail.com), L.V.H.M (lherrera@unizar.es) , M.J.R.Y (mjrodyol@unizar.es)

[5] Department of Chemical Engineering, University of Zaragoza, Campus Río Ebro-Edificio I+D, C/Poeta Mariano Esquillor S/N, 50018 Zaragoza, Spain; M.P (monicapaesamorales@gmail.com)

[6] Institute of Nanoscience and Materials of Aragon (INMA), CSIC-University of Zaragoza, 50009 Zaragoza, Spain.

[7] Aragon Health Research Institute (IIS Aragon), 50009 Zaragoza, Spain.

[8] Department of Molecular Medicine, School of Medicine, University of Colima, 28040 Colima, Mexico; M.D.M (karla_machuca@ucol.mx), O.B.G (oscar.beas.11@gmail.com)

[9] Agri-Food Institute of Aragon IA2, CITA, University of Zaragoza, 50013 Zaragoza, Spain.

[10] Biomedical Research Network Center for the Pathophysiology of Obesity and Nutrition (CIBEROBN), Carlos III Health Institute, 28029 Madrid, Spain.

* Correspondence: M.J.R.Y (mjrodyol@unizar.es), P.F.S (pedromiguel.ferreira@uvigo.gal)

Supplementary Table S1. Retention time (RT), mass spectra (m/z of the ions), and calibration curves of identified phenolic compounds.

Phenolic compound	Retention Time	Precursor Ion (m/z)	Quantifier Ion (m/z)	Qualifier Ion (m/z)	Ionization Mode	Calibration curve equation	R^2
Gallic acid	4.0	169	124	79	Negative	y=252.6x+1271.4	0.9996
3,4-Dihydroxibenzoic acid	8.2	137	93	—	Negative	y=6329.2x-16856.2	0.9995
4-Hydroxibenzoic acid	9.6	137	93	—	Negative	y=22128.0x-25810.8	0.9996
Vanillic acid	10.1	167	108	152	Negative	y=1238.9x+35.3	0.9995
Rutin	10.7	609	270	299	Negative	y=661.5x+40.98	0.9991
p-Coumaric acid	11.0	163	119	—	Negative	y=223.2x+944.4	0.9996

Supplementary Table S2. Characteristics of primers used in RT-qPCR according to MIQE guidelines.

Gene symbol	Accession	Sequence (5′ → 3′)		Amplicon length (nt)	Exons	[Primer] nM	Efficiency %
GAPDH	NM_002046.7	Sense<br>Antisense	CATGTTCGTCATGGGTGTGAACCA<br>AGTGATGGCATGGACTGTGGTCAT	137	6-7	150	95
IL6	NM_000600.5	Sense<br>Antisense	TTCGGTCCAGTTGCCTTCTC<br>CAGCTCTGGCTTGTTCCTCA	403	2-4	-	-
IL8	NM_000584.4	Sense<br>Antisense	CCAGGAAGAAACCACCGGAA<br>TTCTCAGCCCTCTTCAAAAACT	337	1-4	80	95
NOS2	NM_000625.4	Sense<br>Antisense	CCCGAGTCAGAGTCACCATC<br>GGCAGCTCAGCCTGTACTTA	135	14-15	100	96
PTGS2	NM_000963.4	Sense<br>Antisense	ACCCACTCCAAACACAGTGC<br>AAGGGAGTCGGGCAATCATC	243	3-4	100	93
